# An α-Gal-containing neoglycoprotein-based vaccine partially protects against murine cutaneous leishmaniasis caused by *Leishmania major*

**DOI:** 10.1371/journal.pntd.0006039

**Published:** 2017-10-25

**Authors:** Eva Iniguez, Nathaniel S. Schocker, Krishanthi Subramaniam, Susana Portillo, Alba L. Montoya, Waleed S. Al-Salem, Caresse L. Torres, Felipe Rodriguez, Otacilio C. Moreira, Alvaro Acosta-Serrano, Katja Michael, Igor C. Almeida, Rosa A. Maldonado

**Affiliations:** 1 Department of Biological Sciences, Border Biomedical Research Center, the University of Texas at El Paso, El Paso, Texas, United States of America; 2 Department of Chemistry, Border Biomedical Research Center, the University of Texas at El Paso, El Paso, Texas, United States of America; 3 Department of Parasitology, Liverpool School of Tropical Medicine, Pembroke Place, Liverpool, United Kingdom; 4 Laboratório de Biologia Molecular e Doenças Endêmicas, Fundação Oswaldo Cruz (Fiocruz), Rio de Janeiro, Rio de Janeiro, Brazil; 5 Department of Vector Biology, Liverpool School of Tropical Medicine, Pembroke Place, Liverpool, United Kingdom; Fundaçao Oswaldo Cruz, BRAZIL

## Abstract

**Background:**

Protozoan parasites from the genus *Leishmania* cause broad clinical manifestations known as leishmaniases, which affect millions of people worldwide. Cutaneous leishmaniasis (CL), caused by *L*. *major*, is one the most common forms of the disease in the Old World. There is no preventive or therapeutic human vaccine available for *L*. *major* CL, and existing drug treatments are expensive, have toxic side effects, and resistant parasite strains have been reported. Hence, further therapeutic interventions against the disease are necessary. Terminal, non-reducing, and linear α-galactopyranosyl (α-Gal) epitopes are abundantly found on the plasma membrane glycolipids of *L*. *major* known as glycoinositolphospholipids. The absence of these α-Gal epitopes in human cells makes these glycans highly immunogenic and thus potential targets for vaccine development against CL.

**Methodology/Principal findings:**

Here, we evaluated three neoglycoproteins (NGPs), containing synthetic α-Gal epitopes covalently attached to bovine serum albumin (BSA), as vaccine candidates against *L*. *major*, using α1,3-galactosyltransferase-knockout (α1,3GalT-KO) mice. These transgenic mice, similarly to humans, do not express nonreducing, linear α-Gal epitopes in their cells and are, therefore, capable of producing high levels of anti-α-Gal antibodies. We observed that Galα(1,6)Galβ-BSA (NGP5B), but not Galα(1,4)Galβ-BSA (NGP12B) or Galα(1,3)Galα-BSA (NGP17B), was able to significantly reduce the size of footpad lesions by 96% in comparison to control groups. Furthermore, we observed a robust humoral and cellular immune response with production of high levels of protective lytic anti-α-Gal antibodies and induction of Th1 cytokines.

**Conclusions/Significance:**

We propose that NGP5B is an attractive candidate for the study of potential synthetic α-Gal-neoglycoprotein-based vaccines against *L*. *major* infection.

## Author summary

Despite a worldwide prevalence, cutaneous leishmaniasis (CL) remains largely neglected, with no prophylactic or therapeutic vaccine available. In the Old World, CL is mainly caused by either *Leishmania major* or *L*. *tropica* parasites, which produce localized cutaneous ulcers, often leading to scarring and social stigma. Currently, the disease has reached hyperendemicity levels in the Middle East due to conflict and human displacement. Furthermore, the first choice of treatment in that region continues to be pentavalent antimonials, which are costly and highly toxic, and current vector control measures alone are not sufficient to stop disease transmission. Hence, a vaccine against CL would be very beneficial. Previous studies have demonstrated that sugars are promising vaccine candidates against leishmaniasis, since most parasite species have a cell surface coat composed of immunogenic sugars, including linear α-galactopyranosyl (α-Gal) epitopes, which are absent in humans. Here, we have developed an α-Gal-based vaccine candidate, named NGP5B. When tested in transgenic mice which like humans lack α-Gal epitopes in their cells, NGP5B was able to induce a significant partial protection against *L*. *major* infection, by significantly reducing mouse footpad lesions and parasite burden. Altogether, we propose NGP5B as a promising preventive vaccine for CL caused by *L*. *major*.

## Introduction

Cutaneous leishmaniasis (CL) is a neglected, vector-borne disease caused by several species of the protozoan parasite, *Leishmania*. CL currently affects up to 1.2 million people worldwide annually [1], although due to stigma and psychological burden post-infection, its prevalence has been recently estimated to be around 40 million [2]. Because of conflict and large human displacement, the disease is currently hyperendemic in the Middle East [3, 4]. Spread by the bite of an infected sandfly, CL is characterized by localized cutaneous ulcers with different clinical presentations [5]. Currently, there is no commercially available preventive or therapeutic human vaccine for CL and, depending on the region, vector control is insufficient or nonexistent. Furthermore, when prevention fails, infected patients rely on expensive and highly toxic drugs (mainly antimonials), which are costly and have become ineffective in some areas due to emerging resistant parasite strains [6]. Despite current advances, the development of a safe, affordable and fully protective anti-leishmaniasis vaccine continues to be a challenge. Experimental vaccines have been explored; however, they either lack the ability to confer full sterile protection against parasite challenge, show outcomes which considerably differ by location, and/or the clinical trial was abandoned due to safety regulations [7–9]. Therefore, development of a fully protective vaccine against CL is much needed.

During the life cycle of *L*. *major* or *L*. *tropica*, the main species responsible for CL in the Old World, parasites must survive and proliferate inside the hostile environment of human macrophages. They use different evasion mechanisms, including the formation of a protective glycocalyx barrier that is mainly composed of protein-free glycosylphosphatidylinositol (GPI) anchors, known as glycoinositolphospholipids (GIPLs), among other surface glycoconjugates [10–14]. In *L*. *major*, highly abundant Type-II GIPL-2 and GIPL-3 are capped with terminal, non-reducing α-galactopyranosyl (α-Gal*p* or α-Gal) residues with different structural configurations (Gal*p*α(1,3)Gal*ƒ*β-R and Gal*p*α(1,6)Gal*p*α(1,3)Gal*ƒ*β-R, respectively; where R is the remaining GPI anchor) [15]. These glycolipids are conserved throughout the parasite’s life cycle, and are highly abundant (i.e., >10^10^ copies/cell) in the amastigote (mammalian) stage [13]. Due to the inactivation of the α1,3-galactosyltransferase (α1,3GalT) gene [16], terminal, non-reducing and linear α-Gal epitopes are absent and, therefore, highly immunogenic to humans and Old World nonhuman primates. Thus, healthy human individuals normally produce anti-α-Gal antibodies (also known as normal anti-Gal antibodies) against α-Gal epitopes found in lipopolysaccharides of enterobacteria [17–19]. On the other hand, during infections with *Trypanosoma cruzi*, *Leishmania* spp., or *Plasmodium* spp., parasite-specific anti-α-Gal antibodies are elicited [20–27]. These antibodies have different specificities than normal anti-α-Gal antibodies. For instance, *T*. *cruzi*-specific anti-α-Gal antibodies have been used as reliable biomarkers for diagnosis and follow-up of chemotherapy of patients with chronic Chagas disease [28, 29]. More recently, similar roles and applications have been proposed for anti-α-Gal antibodies produced during CL [26]. In both acute and chronic phases of Chagas disease, *T*. *cruzi*-specific IgM and IgG anti-α-Gal antibodies are trypanolytic and protective [22, 25, 30], and a vaccine based on the major parasite α-Gal epitope has been proposed [31]. In *Plasmodium* infections in children, however, IgM but not IgG anti-α-Gal antibodies appear to have a protective effect [27]. Although the levels of anti-α-Gal antibodies are very high in *Leishmania* infections [21, 23, 24, 26], it has not yet been demonstrated whether these antibodies have any protective role. More recently, Jaurigue and Seeberger [32] have proposed α-Gal epitopes as vaccine candidates against *Leishmania* spp. and other major protozoan parasites. In this study, we explore this possibility by evaluating neoglycoproteins (NGPs) containing synthetic α-Gal epitopes in different configurations, as potential preventive *L*. *major* vaccine candidates in the α1,3-galactosyltransferase-knockout (α1,3-GalT-KO) mouse model, which does not express terminal α-Gal epitopes on their cells [33, 34].

## Materials and methods

### Ethics statement

Animal procedures were performed according to NIH guidelines and the appropriate protocol (A-201209-1) approved by UTEP’s Institutional Animal Care and Use Committee (IACUC).

The Institutional Review Board (IRB) protocol was approved by IRB Committee of the Liverpool School of Tropical Medicine (LSTM) (Research Protocol 15.037). Written informed consent was obtained from all participants. Only adults participated in the study. For the collection of human samples, ethical approval (12·06R) was granted by both the Liverpool School of Tropical Medicine and the KSA Ministry of Health Ethical Committees.

### Mice

C57BL/6 α1,3-Galactosyltransferase-knockout (α1,3GalT-KO) mice [33, 34] were kindly donated by Prof. Peter J. Cowan, St. Vincent’s Hospital Melbourne and University of Melbourne, Australia. Animals were bred and maintained under biosafety level 2 (BSL-2), pathogen-free conditions at the Laboratory Animal Resources Center (LARC) at UTEP. Six to eight-week old female α1,3GalT-KO mice were used for all experiments.

### Human sera

Human serum samples from adult patients with *L*. *major* active (n = 5) or cured CL (n = 5) infections, or adults with heterologous (non-CL) infections (n = 5) were obtained from the Al-Ahsa region, Kingdom of Saudi Arabia (KSA), which is exclusively endemic for *L*. *major* CL [35]. Detailed information on each serum sample regarding sex, age, and race of the patient was not available for this study.

### Parasites

Promastigote forms of transgenic *L*. *major* expressing firefly luciferase (*L*. *major*-*luc*) (Lmj-FV1-LUC-TK [*L*. *major* strain Friedlin], clone V1) [36, 37], kindly donated by Prof. Stephen M. Beverley (Washington University in St. Louis, MO), were grown at 28°C in M199 medium supplemented with 10% heat-inactivated fetal bovine serum (FBS) (Gibco, Thermo Fisher Scientific, Waltham, MA) and 1% penicillin-streptomycin. Infective metacyclic promastigote forms were purified by Ficoll gradient at stationary phase (3–5 days), as previously described [38].

### Neoglycoproteins

**Fig 1:**
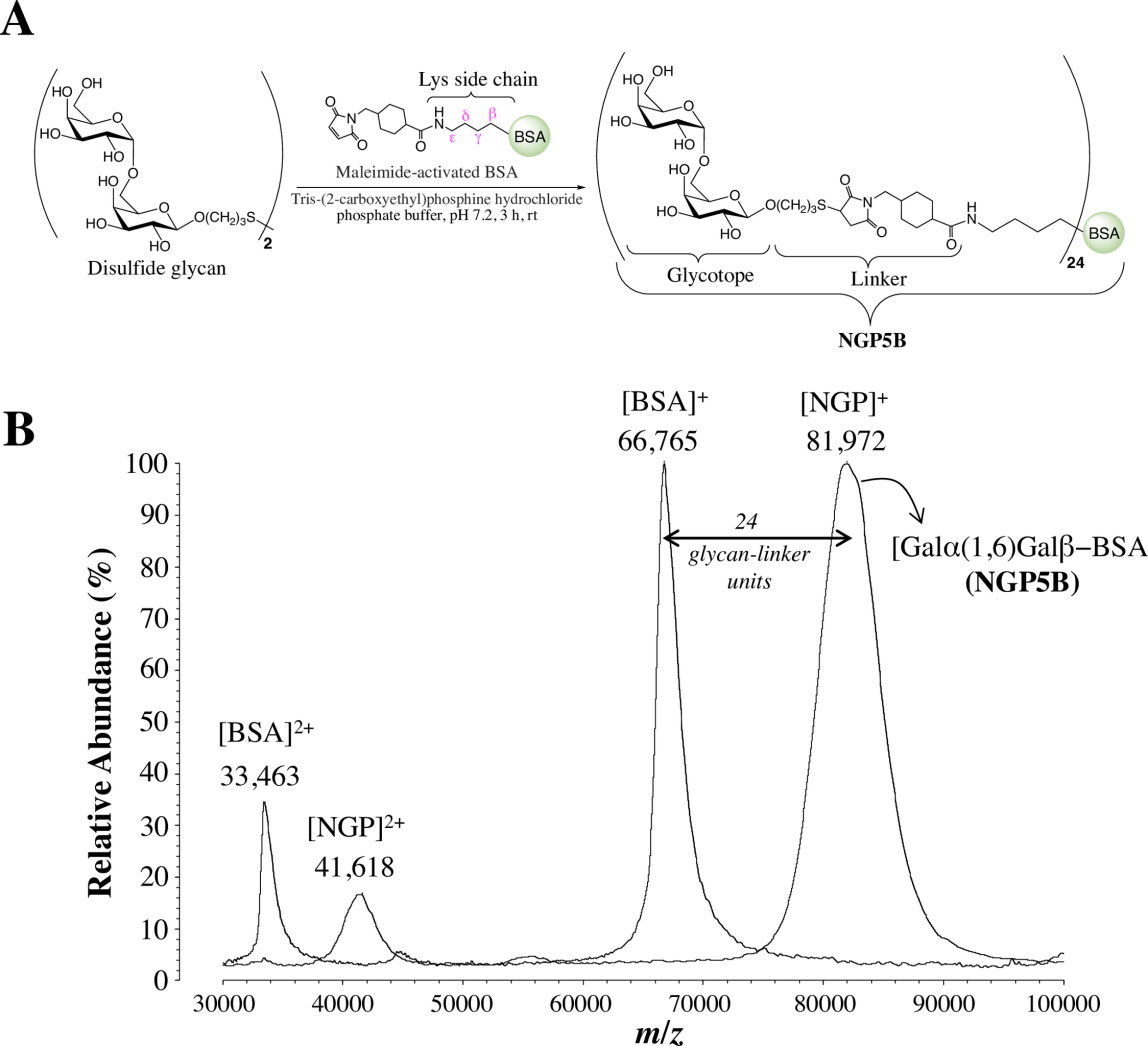
Schematic representation of NGP synthesis. (**A**) Conjugation of mercaptopropyl saccharide derivative (obtained by reduction of the disulfide glycan *in situ*) to maleimide-activated BSA to produce the NGP Galα(1,6)Galβ-BSA (NGP5B). For simplification, the linker and lysine side chain are omitted from all NGP nomenclatures used in this study. (**B**) MALDI-TOF-MS analysis of BSA before and after maleimide-derivatization and glycan coupling to generate NGP5B. Doubly-charged ([BSA]^2+^ and [NGP]^2+^) and singly-charged ([BSA]^+^ and [NGP]^+^) ions of BSA and NGP are indicated. Lys, lysine.

The NGPs Galα(1,3)Galβ(1,4)Glcβ-BSA (NGP1B), Galα-BSA (NGP3B), Galα(1,6)Galβ-BSA (NGP5B), Galα(1,2)Galβ-BSA (NGP8B), Galα(1,6)[Galα(1,2)]Galβ-BSA (NGP11B), Galα(1,4)Galβ-BSA (NGP12B), Galβ-BSA (NGP13B), and Galα(1,3)Galα-BSA (NGP17B) were obtained as previously described [39]. Briefly, mercaptopropyl glycan derivatives were synthesized and covalently coupled to commercially available maleimide-activated bovine serum albumin (BSA) (Pierce, Thermo Fisher Scientific) to generate their respective NGPs. A schematic representation of the NGP synthesis is shown in Fig 1A.

The glycan load for each NGP was measured using matrix-assisted laser desorption/ionization time-of-flight mass spectrometry (MALDI-TOF-MS) (Axima, Shimadzu), as described [31]. Lyophilized underivatized BSA (control) or NGP was first diluted in sterile deionized water to a 1.0–1.5 mg/μL solution. Pre-loading mix was prepared by adding 5 μL of diluted NGP or BSA, 2.5 μL of sinapinic acid (Sigma-Aldrich), and 1.5 μL acetonitrile:water (2:1, v/v) with 0.1% trifluoroacetic acid (Thermo Fisher Scientific). Next, 0.5 μL of pre-loading mix was applied to MALDI-plate spot and allowed to air dry. Once dried, 100 shots were fired per spot and 20 spectra were acquired. The glycan load per NGP molecule was calculated by subtracting the BSA average molecular mass from the NGP average molecular mass, and dividing it by the nominal molecular mass of one glycan-linker unit (636 Da) (Fig 1B).

### Immunizations

The NGPs Galα(1,6)Galβ-BSA (NGP5B), Galα(1,4)Galβ-BSA (NGP12B), and Galα(1,3)Galα-BSA (NGP17B), obtained as described above, were first redissolved in sterile deionized water and then diluted in sterile phosphate-buffered saline (PBS), pH 7.4, immediately prior to immunizations. Phosphorothioate-modified oligodeoxynucleotide (ODN) sequence 1826 containing two CpG motifs (5’-TCCATGACGTTC-CTGACGTT-3’) (CpG ODN or CpG adjuvant) was obtained from InvivoGen (San Diego, CA) and was dissolved in endotoxin-free, 0.9% NaCl solution, at 2 mg/mL. NGP12B, NGP17B, and NGP5B were administrated at 10 μg/dose in a final volume of 200 μL PBS. NGP5B was also administrated at 10 μg/dose in combination with CpG (NGP5B+CpG) adjuvant at 20 μg/dose in a final volume of 200 μL. Control groups were immunized with CpG (20 μg/dose) or PBS alone in a final volume of 200 μL. A total of four immunizations were subcutaneously administrated at 7-day intervals.

### Chemiluminescent enzyme-linked immunosorbent assay (chemiluminescent ELISA) with CL-infected patients

Chemiluminescent ELISA [26] was performed for screening the IgG response to various α-Gal-containing NGPs in patients with active or cured CL infections, or patients with heterologous (non-CL) skin infections. Briefly, 96-well Nunc polystyrene microplates (Thermo Scientific) were coated overnight at 4°C with 250 ng/well of each NGP in 200 mM carbonate-bicarbonate buffer, pH 9.6 (CBB). Plates were blocked with 100 μL 1% BSA-PBS (BSA, Sigma-Aldrich, St. Louis, MO) for 1 h at 37°C before washing three times with PBS-0.05% Tween 20 (Sigma-Aldrich) (PBS-T). Pools of human sera at 1:100 dilution (in PBS) were analyzed in triplicate. Plates were incubated for 1 h at 37°C. Goat anti-human IgG antibody (Sigma-Aldrich; 50 μL, 1:1000 dilution) in PBS was added and incubated for 1 h at 37°C. Donkey biotinylated anti-goat IgG conjugate (Thermo Fisher Scientific; 50 μL, 1:1000 dilution) in PBS was added and plates were incubated for 1 h at 37°C. Horseradish peroxidase (HRP)-streptavidin (Invitrogen; 50 μL, 1:2000 dilution) in PBS was added and plates were incubated for 30 min at 37°C. Plates were washed three times between steps with PBS-T, except before blocking. The reaction was developed with Super-Signal Chemiluminescent Substrate (Thermo Fisher Scientific) at 1:8 dilution in 50 μL CBB and relative luminescence units were measured using a FLUOstar Omega microplate reader (BMG LabTech, Baden-Württemberg, Germany). Serum samples used to generate the pools were selected at random from three different patient groups: cured CL infections, active CL infections, or heterologous diseases (control group). Heterologous controls included patients that had skin abnormalities that mimicked CL (e.g., eczema) but without the *Leishmania* parasite. Wells with PBS (no serum controls) were added to address background nonspecific reactivities.

### Chemiluminescent ELISA with sera from vaccinated α1,3GalT-KO mice

Blood was obtained from all animals by facial vein puncture, and serum was obtained by centrifugation (2,500 x*g*, 10 min, 4^°^C). To assess the humoral immune response, levels of specific anti*-*α*-*Gal antibody titers were determined by chemiluminescent ELISA, as previously described [26, 39]. MaxiSorp Nunc polystyrene microplates (Thermo Fisher Scientific) were coated with 125 ng/well of each NGP in CBB and incubated overnight at 4°C. Plates were blocked with 200 μL 1% BSA-PBS, and incubated with mouse sera (50 μL) from vaccinated or control groups at 1:100 dilution for 1 h at 37^°^C. Donkey biotinylated anti-mouse (Pierce, Thermo Fisher Scientific) was used at 1:2000 dilution in 1% BSA/PBS with 0.05% Tween 20 (1% BSA/PBS-T). NeutrAvidin-HRP (Pierce, Thermo Fisher Scientific) was used at 1:5000 dilution in 1% BSA/PBS-T. Plates were washed 3x between steps with PBS-T, except before blocking. The reaction was developed with SuperSignal Pico Chemiluminescent Substrate (Pierce, Thermo Fisher Scientific). Luminescence (in RLUs) was measured by Luminoskan luminometer (Thermo Fisher Scientific).

### α-Galactosidase treatment

To test the specificity of the IgG antibodies elicited in mice immunized with NGP5B or NGP5B+CpG, immobilized NGP5B used as antigen was pretreated with green coffee bean α-galactosidase (G8507, Sigma-Aldrich), as previously described [26]. First, MaxiSorp Nunc polystyrene microplate (Thermo Fisher Scientific) wells were coated with 125 ng/well of NGP5B in CBB and incubated overnight at 4°C. The plate was blocked with 200 μL 5% skim milk-PBS for 1 h at 37^°^C, and washed three times with 200 μL PBS-T. In parallel, two hundred microliters of α-galactosidase (in ammonium sulfate suspension, ≥9 units/mg protein) were centrifuged at 10,000 xg for 10 min at 4°C to remove the excess ammonium sulfate. The supernatant was discarded and the pellet containing the enzyme was gently redissolved in ice-cold 100 mM potassium phosphate buffer (pH 6.5). Fifty microliters of the enzyme solution (0.002 units/μL) were added to each well, and the plate was incubated for 24 h at 37°C. The microplate was washed twice with 200 μL PBS-T and the chemiluminescent ELISA was performed as described above.

### Parasite challenge

Six to eight-week old female α1,3GalT-KO mice (n = 3 per group) were inoculated with 1 x 10^5^ *L*. *major*-*luc* metacyclic promastigotes in the left hind footpad 3 weeks after last immunization. Mice lesion size (mm) was monitored weekly with a digital caliper.

### *In vivo* bioluminescence imaging and weight monitoring

To measure disease progression, *in vivo* bioluminescence imaging of *L*. *major*-*luc* was acquired. Mice were injected intraperitoneally (i.p.) with 150 mg.kg^-1^ D-luciferin (Gold Biotechnology, St. Louis, MO) and anesthetized with 2.5% gaseous isoflurane in oxygen. Images were acquired 15 min after luciferin injection using IVIS Lumina III *In Vivo* Imaging System (PerkinElmer, Waltham, MA). Additionally, for any sign of toxicity mice were periodically weighed, and weight change was normalized using the initial mouse weight.

### Parasite load by quantitative PCR

At experimental endpoint, mice were sacrificed by CO_2_ overdose and infected footpads from all groups were harvested. Genomic DNA from 20 to 30 mg of tissue was extracted with High Pure PCR Template Preparation Kit following the manufactures protocol without modifications (Roche Molecular Systems, Indianapolis, IN). As internal control, a linearized pUC57 plasmid containing a sequence from *Arabidopsis thaliana* was spiked before all DNA extractions as previously described [40]. Parasite load was measured by absolute quantification based on a standard DNA curve ranging from 0.5 to 10^5^ *L*. *major* parasite equivalents/mL. A standard curve was produced by extracting DNA from a 20 to 30 mg tissue fragment, spiked with 10^5^ *L*. *major* promastigotes (spiked DNA). In parallel, DNA was extracted from 20 to 30 mg of tissue fragment from uninfected mice (negative control DNA). Afterwards, spiked DNA was 10-fold serially diluted in the negative control DNA. Amplification of 120 bp-fragment from the kinetoplast DNA of *L*. *major* was performed using 100 nM of forward primer (5’-CTTTTCTGGTCCTCCGGGTAGG-3’), 100 nM of reverse primer (5’-CCACCCGGCCCTATTTTACACCAA-3'), and 50 nM of TaqMan probe (FAM-TTTTCGCAGAACGCCCCTACCCGC-TAMRA); a total of 100 ng of DNA was added to a reaction in a final volume of 20 μL [41, 42]. The exogenous internal amplification control (IAC) was amplified using 100 nM of forward primer (5’-ACCGTCATGGAACAGCACGTA-3’), 100 nM of reverse primer (5’- CTCCCGCAACAAACCCTATAAAT-3’), and 50 nM of TaqMan probe (VIC-AGCATCTGTTCTTGAAGGT-NFQ-MGB). PCR conditions consisted of 50°C for 2 min, 94°C for 10 min, followed by 45 cycles at 94°C for 15 sec and 55°C for 1 min. Samples were run in triplicate in Step One Plus Real Time PCR System (Applied Biosystems).

### Serum cytokine profile

Th1, Th2, and Th17 cytokines (IL-12p40, IL-2, IFN-γ, TNF-α, IL-5, IL-4, IL-10, IL-6, and IL-17) were analyzed using multiplex kit, MILLIPLEX Mouse Cytokine Magnetic Bead Panel (EMD Millipore, Billerica, MA). Sera (at 1:2 dilution) from all immunized groups and controls were analyzed at 3 weeks after last immunization, prior to challenge (day 0), following the manufacturer’s protocol.

### Immunoglobulin isotyping

Additionally, antibody levels of mouse IgG1, IgG2a, IgG2b IgG3, and IgE specific to NGP5B were analyzed by ELISA at boost 3 (B3, day -21) and experimental endpoint (day 43). Briefly, 96-well Nunc polystyrene microplates (Thermo Fisher Scientific) were coated overnight at 4°C with 125 ng/well of NGP5B in CBB. Plates were blocked with 200 μL 1% BSA-PBS for 1 h at 37°C. Mouse serum was diluted at either 1:400 (B3) or 1:100 (endpoint) dilution in PBS. To detect the various mouse IgG subtypes secondary antibodies conjugated to HRP were used (goat anti-mouse IgG1-HRP, goat anti-mouse IgG2a-HRP, goat anti-mouse IgG2b-HRP, and goat anti-mouse IgG3-HRP). All secondary antibody conjugates were from Abcam (Cambridge, MA). The antibodies were diluted 1:2000 in PBS and 100 μL was added to each well. Plates were incubated for 1 h at 37°C, followed by washing three times with PBS-T (200 μL) between the steps. The reaction was developed with 100 μL Super-Signal Chemiluminescent Substrate (Thermo Fisher Scientific) at1:8 dilution in CBB and read using a FLUOstar Omega microplate reader (BMG LabTech). A pool of sera from α1,3GalT-KO mice was used as negative control. Biological replicates were run for each test group.

### Lytic assay of *L*. *major* metacyclic promastigotes

*L*. *major* metacyclic promastigotes (200 μL of 1 x 10^6^ parasites/ml DMEM) were pre-incubated with 10% fresh sera (with active complement) from mice immunized with NGP5B, NGP5B+CpG, CpG, or PBS. Prior to the lytic assay, in selected incubation mixtures, complement was heat-inactivated at 56°C for 30 min before adding serum to the parasites. 0.5 μg/mL propidium iodide (PI; Sigma-Aldrich) diluted in deionized water was added after 10 min of parasite-serum incubation, followed by additional 30-min incubation at room temperature (RT) for all experimental samples. A total of 5,000 events were analyzed by flow cytometry using a Gallios Flow Cytometer (Beckman Coulter). The percentage of PI-positive events (= % of dead parasites) were graphed.

### CD4^+^ and CD8^+^ T cell analysis by flow cytometry

Three weeks after the last immunization prior to challenge (day 0) or post-challenge (endpoint, day 43), splenocytes were harvested from mice (n = 3 per group) and cultured in freshly prepared Dulbecco’s modified Eagles’ medium (DMEM), supplemented with 10% heat-inactivated FBS (Gibco, Thermo Fisher Scientific), 1% penicillin-streptomycin, and 0.5 mM 2-mercaptoethanol (2-ME). After splenocyte harvesting, ten milliliters of red blood cell lysis solution (0.83% ammonium chloride, 0.1% potassium bicarbonate, 0.04% EDTA, pH 7.4), was added to cells for 10 min, and splenocytes were cultured in 12-well flat-bottom plates (Corning Costar, Thermo Fisher Scientific) and stimulated *in vitro* with 20 μg/ml of antigen (NGP5B or NGP5B+CpG) at 37°C, in 5% CO_2_ atmosphere, for 24 h. In brief, Fc-gamma receptor (FcγR) was blocked with 10% heat-inactivated naïve α1,3GalT-KO mouse serum and cells were stained with fluorochrome-conjugated antibodies PE-Cy7-labeled anti-CD3e, PE-labeled anti-CD4, FITC-labeled anti-CD8, APC-labeled anti-CD44, and Alexa Fluor 700-labeled anti-CD69 (all conjugates were from BD Bioscience, San Jose, CA), along with the appropriate isotype controls (BD Bioscience) for 30 min at 4°C. Cells were washed with PBS with 1% BSA plus 0.09% sodium azide, and fixed with 1% paraformaldehyde. A total of 10,000 events were acquired using a Gallios Flow Cytometer (Beckman Coulter) and analyzed by Kaluza Software (Beckman Coulter). Gates were set for cells, followed by lymphocytes (CD3e-Pe-Cy7 labeling) using forward and side scatter properties, and the frequencies and percentages of activated CD4^+^ and CD8^+^ T cells were obtained on CD3^+^ T cells.

### Statistical analysis

Each data point is presented as average of triplicate determinations with their corresponding standard error of the mean (S.E.M.). Student *t-*test, One-way ANOVA, or Two-way ANOVA were employed in the statistical analysis. Graphs and statistical analysis were attained using Graph Pad Prism 6 Software (GraphPad Software, Inc., La Jolla, CA).

## Results

### Evaluation of synthetic neoglycoproteins as potential biomarkers in *L*. *major*-infected individuals

**Fig 2:**
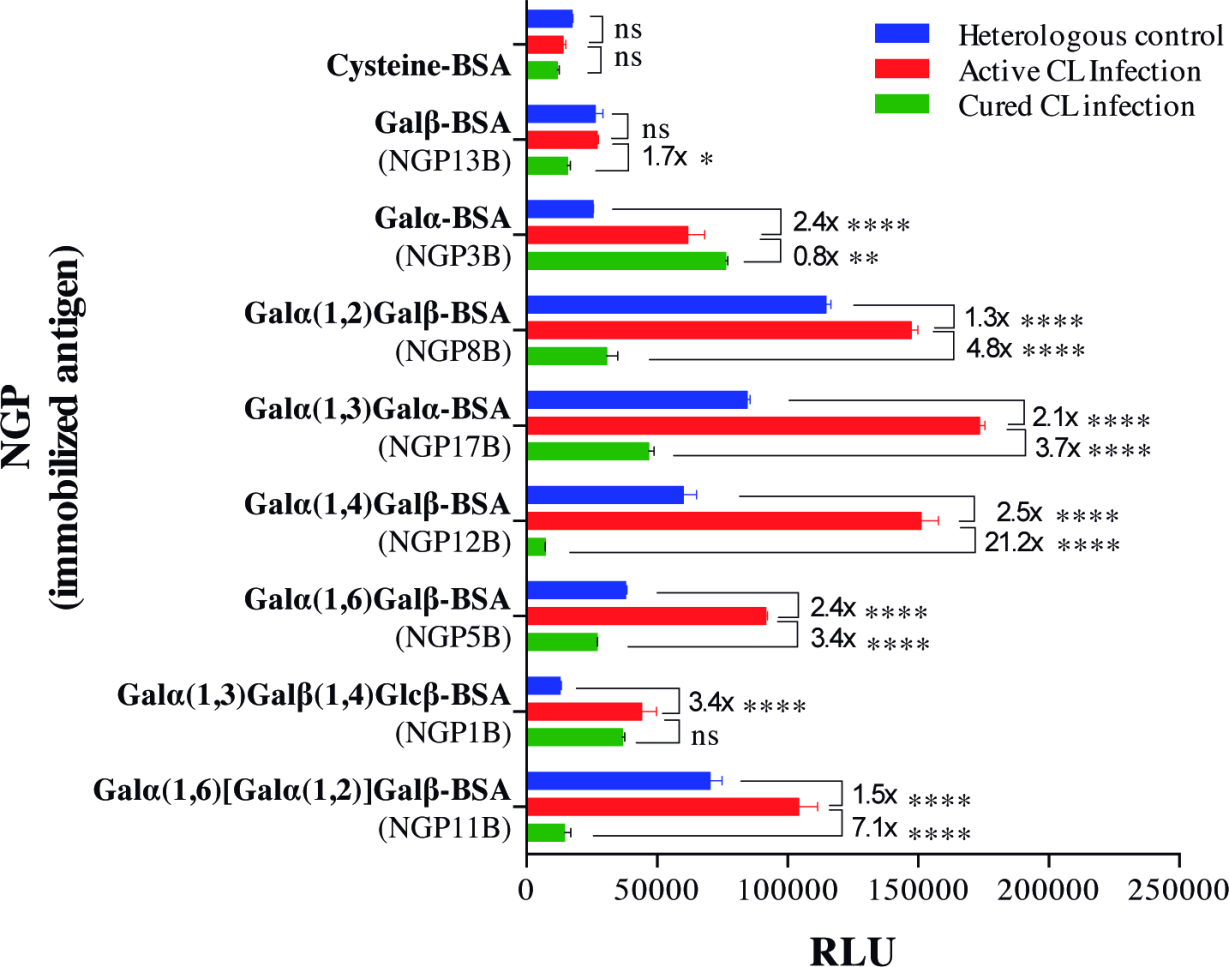
α-Gal-NGPs as biomarkers of active CL in patients with *L*. *major* infection. Assessment by chemiluminescent ELISA of α-Gal-containing NGPs and controls (Cysteine-BSA and Galβ-BSA) were immobilized on a microplate and reacted with pools of sera (at 1:100 dilution) from patients with active or cured CL, or heterologous skin (non-CL) infections (n = 5 per group, randomly selected) from an endemic region (Saudi Arabia), as described [26]. RLU, Relative luminescence units. Error bars indicate S.E.M. of triplicate determinations. The fold difference in reactivity between active CL vs. cured CL, and active CL vs. heterologous infection are indicated. Two-way ANOVA with Tukey’s multiple comparisons: ns, non-significant; (*), *P*<0.05; (**), *P*<0.01; (***), *P*<0.001; (****), *P*<0.0001.

The antigenicity and specificity of eight NGPs containing different synthetic α-Gal epitopes were evaluated by chemiluminescent ELISA using pools of sera from *L*. *major*-infected patients with active or cured CL infections, from an endemic region of Saudi Arabia. Heterologous controls included patients who had skin abnormalities that mimicked CL (e.g., eczema) but without *Leishmania* parasites (Fig 2). NGP17B (Galα(1,3)Galα-BSA**)**, NGP12B (Galα(1,4)Galβ-BSA**)**, and NGP5B (Galα(1,6)Galβ-BSA**)** showed the best differential reactivity between active CL vs. cured CL infection, and active CL vs. heterologous infection. Although highly reactive with the serum pool from patients with active CL infection, NGP8B and NGP11B showed higher cross-reactivity with the heterologous control. Conversely, NGP3B and NGP1B showed little or no differential reactivity between active and cured CL infection. Together, these results suggest that NGP17B, NGP12B, and NGP5B are immunogenic and potential biomarkers of active CL infection.

### Synthetic NGPs as vaccine candidates against *L*. *major* infection

**Fig 3:**
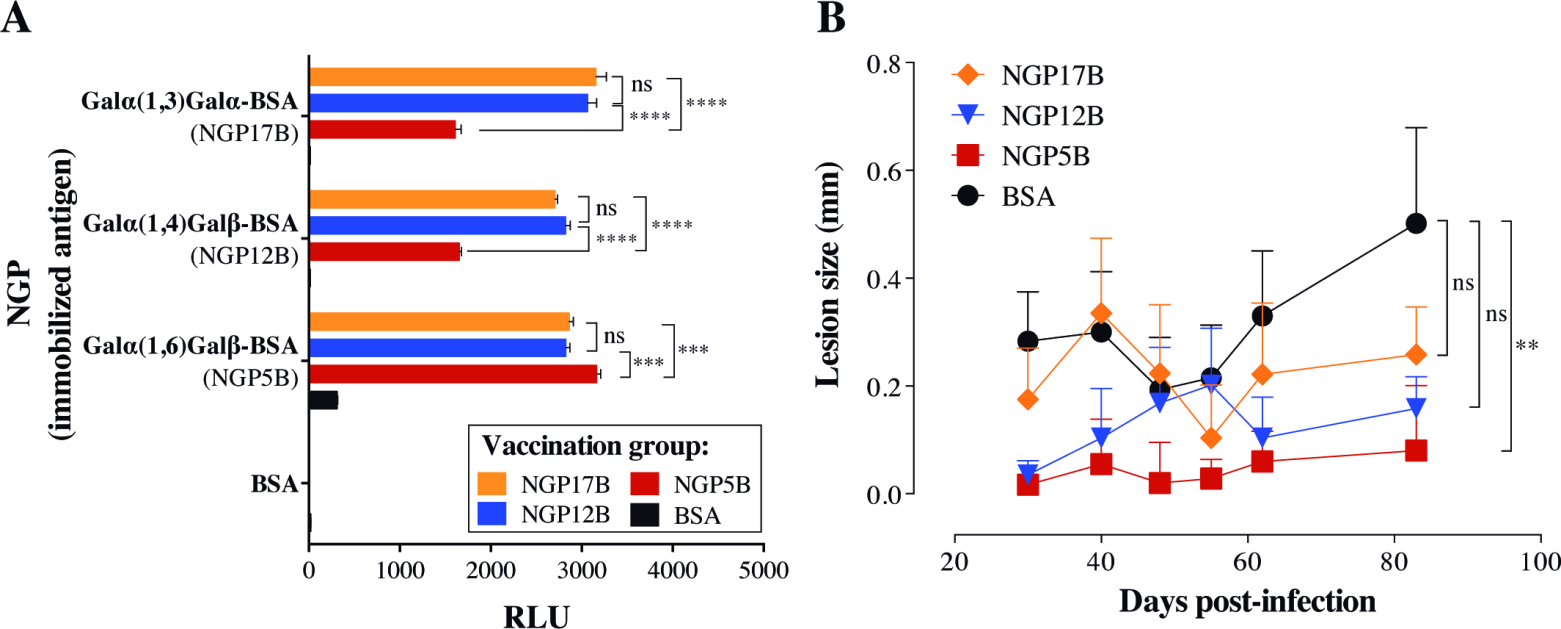
α-Gal-NGPs as potential vaccine candidates against *L*. *major*. (A) Chemiluminescent ELISA to measure anti-α-Gal antibody levels in α1,3GalT-KO mice immunized with the α-Gal-containing NGP17B, NGP12B, or NGP5B. Immunizations groups are indicated in the legend, whereas NGPs and control (BSA) used as antigens in the chemiluminescent ELISA are shown in the Y-axis. Sera were used at 1:100 dilution. Two-way ANOVA with Tukey’s multiple comparisons: ns, non-significant; (***), *P<*0.001; (****), *P<*0.0001. (B) Lesion size (mm) in mice immunized with α-Gal-NGP (NGP12B, NGP17B, or NGP5B) or control (BSA), and then challenged with 1 x 10^5^ *L*. *major-luc* metacyclic promastigotes. One-way ANOVA (compared with BSA control): ns, non-significant; (**), *P<*0.01. (A and B) Error bars indicate S.E.M. of triplicate determinations.

The relative high specificity and antigenicity of NGP12B, NGP17B, and NGP5B in active CL infection led us to interrogate whether these NGPs would also be able to induce protective response against *in vivo L*. *major* infection. To this end, we used C57BL/6 α1,3GalT-KO mice, which lack α-Gal epitopes in their cells and tissues, therefore mimicking human antibody responses to these epitopes [33, 43]. Animals (n = 6 per group) were subjected to four immunizations of NGP12B, NGP17B, or NGP5B, or control (BSA) (10 μg/dose/200 μL PBS, at 7-day intervals). Three weeks after the last immunization, the specific IgG levels were measured by chemiluminescent ELISA, as previously described [31]. A strong antibody response was observed in the animals immunized with NGP12B, NGP17B, or NGP5B when compared to the control group (BSA) (Fig 3A). However, we also observed a significant cross-reactivity between the NGPs, although antibodies against NGP5B had lower (~39–49%) cross-reactivity with NGP12B and NGP17B. Next, to assess the protective ability of these NGPs, mice were challenged with 1 x 10^5^ *L*. *major-luc* metacyclic promastigotes and footpad lesion was evaluated for 81 days post-challenge (Fig 3B). In comparison with control group (BSA), NGP5B vaccination was able to maintain significantly lower lesion size in immunized mice throughout the course of the infection. Although NGP12B and NGP17B showed a protective trend towards the end of infection follow-up, there was no statistical significance when compared to BSA group. Taken together, these results indicated that NGP5B had a higher protective effect than NGP12B and NGP17B, and therefore was selected to be further evaluated as a vaccine candidate.

### NGP5B elicits significant protection against *L*. *major* challenge in α1,3GalT-KO mice

**Fig 4:**
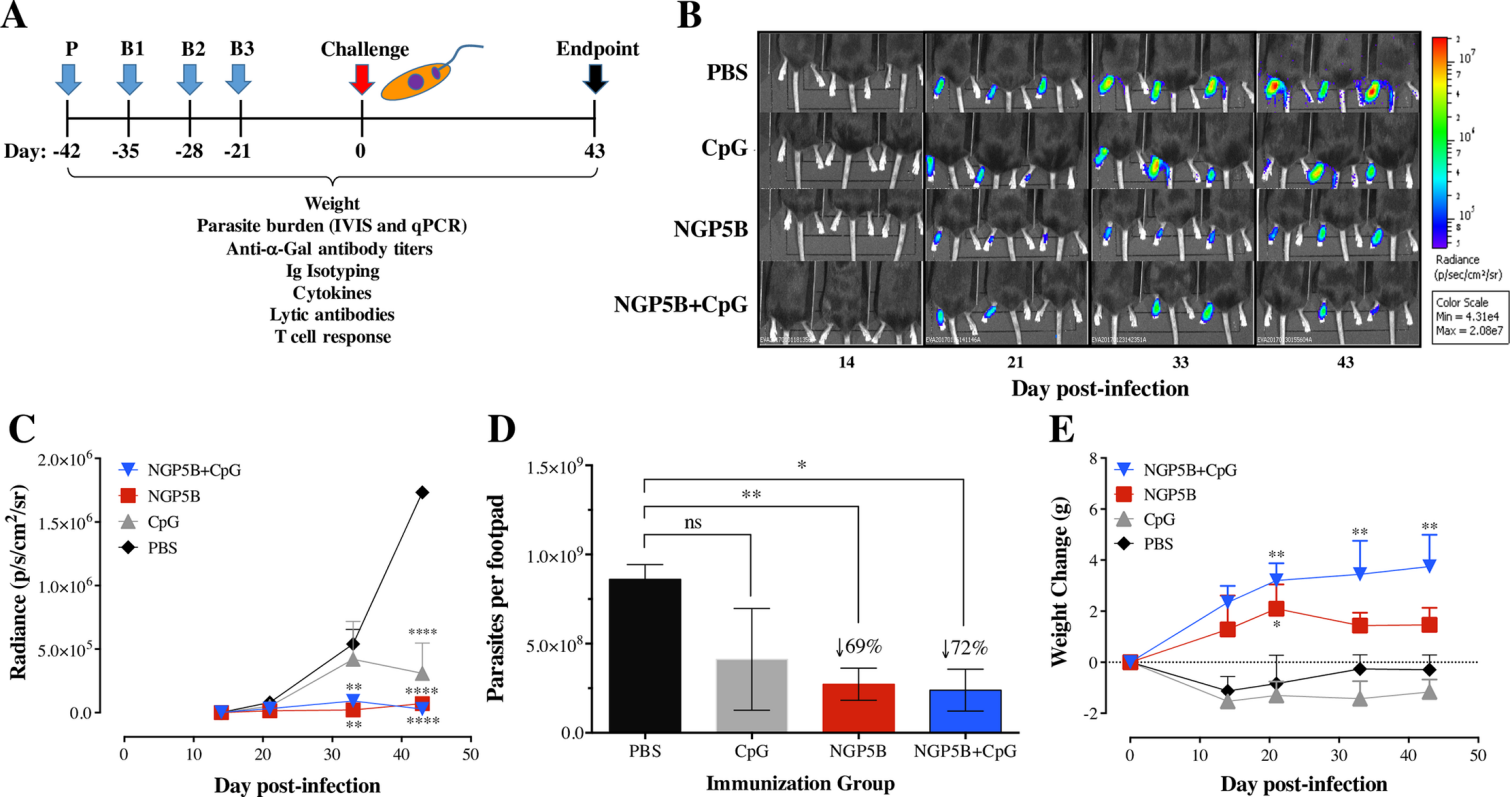
Vaccination with NGP5B or NGP5B+CpG elicits partial but significant protection against *L*. *major* infection in α1,3GalT- KO mice. (A) Vaccination experimental design. Prime (P), boost 1–3 (B1-B3). Three weeks after B3, immunized α1,3GalT-KO mice were challenge with 1 x 10^5^ *L*. *major-luc* metacyclic promastigotes. Experiment was concluded at 43 dpi. (B) *In vivo* bioluminescence imaging of α1,3GalT-KO mice immunized with NGP5B, NGP5B+CpG, CpG or PBS, and then challenged with 1 x 10^5^ *L*. *major-luc* metacyclic promastigotes. Bioluminescence images obtained at 14, 21, 33, and 43 dpi. (C) Quantification of bioluminescence emitted in the footpad by α1,3GalT-KO mice immunized with NGP5B, NGP5B+CpG, CpG, or PBS and challenged with *L*. *major-luc* metacyclic promastigotes. Two-way ANOVA with Dunnett’s multiple comparison test (compared with PBS group): (**), *P* <0.01; (****), *P*<0.0001. (D) Quantification of parasitic load (parasites per footpad) by qPCR, at the experimental endpoint (43 dpi). Two-way ANOVA with Dunnett’s multiple comparison test: ns, non-significant; (*), *P* <0.05; (**), *P*<0.01. (E) Assessment of treatment toxicity established by weight change in α1,3GalT-KO mice immunized with NGP5B, NGP5B+CpG, CpG, or PBS, and then challenged. Two-way ANOVA with Dunnett’s multiple comparison test (compared with PBS group): (*), *P* <0.05; (**), *P*<0.01. (C-E) Error bars indicate S.E.M. of triplicate determinations.

First, six mice per group were immunized with NGP5B (10 μg/dose) or in combination with the adjuvant CpG (20 μg/dose). Control mice groups were immunized with either 20 μg/dose of CpG, or PBS. Prime (P) and 3 boost immunizations (B1-B3) were performed subcutaneously at 7-day intervals, followed by *L*. *major* challenge three weeks after last immunization (B3) (Fig 4A). *In vivo* bioluminescence imaging (n = 3) was acquired at 14, 21, 33, and 43 days post-infection (dpi) (Fig 4B). Quantification of luminescence in the footpads is represented by the radiance (photons per second per centimeter squared per steradian, p/s/cm^2^/sr) per infected footpad/mouse. In comparison to the PBS control group, NGP5B significantly reduced (*P*<0.0001) parasite bioluminescence, when administrated alone or in combination with the CpG adjuvant(*P*<0.0001) (Fig 4C). This was corroborated by significant parasite load reduction in the footpad as detected by qPCR in animals immunized with NGP5B (69%) (*P*<0.01) and NGP5B+CpG (72%) (*P*<0.05), as compared to the PBS control group (Fig 4D). Furthermore, in contrast to animals treated with CpG or PBS, animals vaccinated with NGP5B or, in particular, NGP5B+CpG, gained weight following parasite challenge, indicating therefore that these animals were healthy (Fig 4E). Taken together, these findings confirmed that both NGP5B and NGP5B+CpG induce partial but significant protect against *L*. *major* infection.

### NGP5B induces a specific humoral immune response

**Fig 5:**
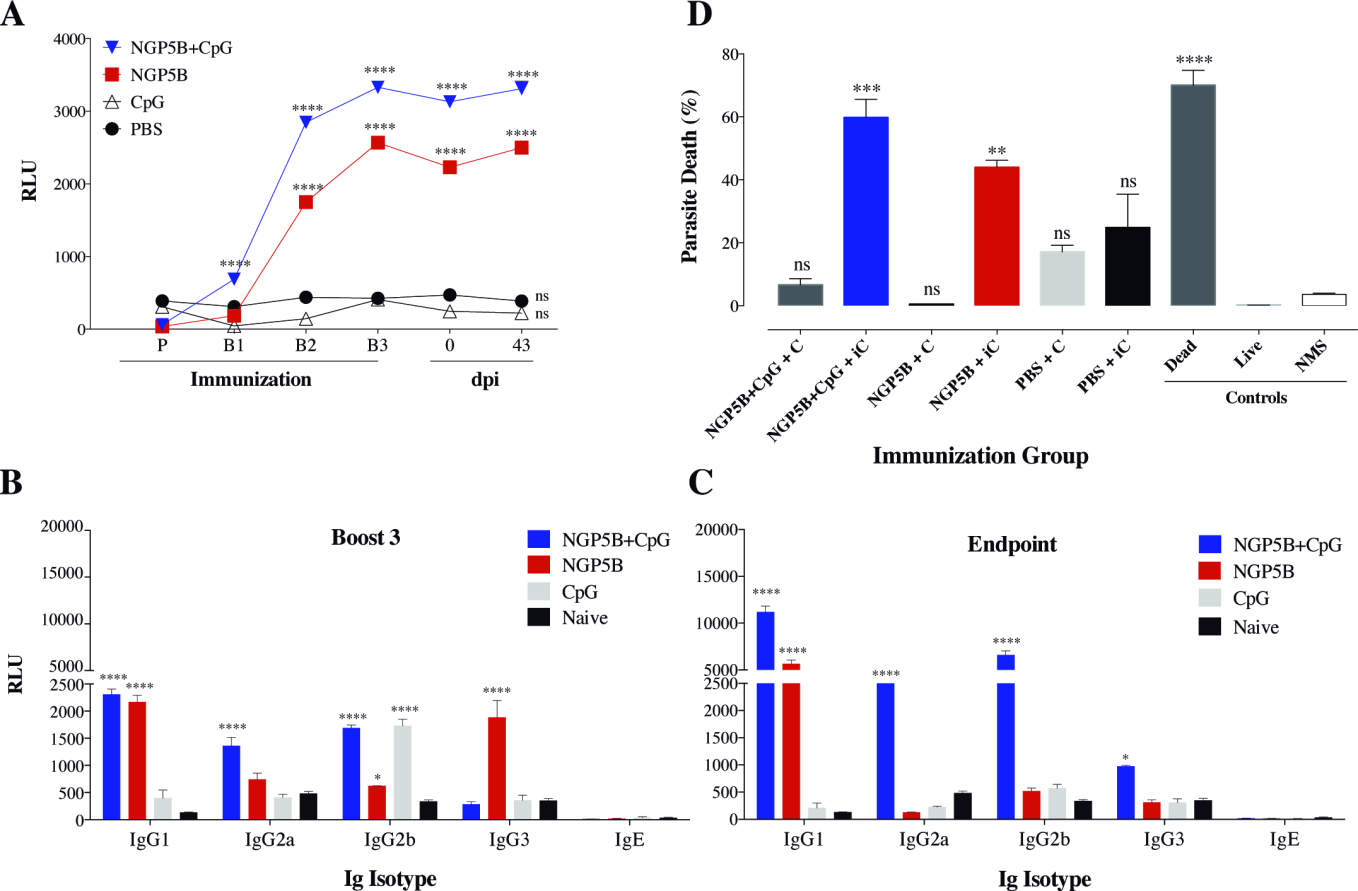
Analysis of humoral immune response of NGP5B immunized mice. (A) Chemiluminescent ELISA reactivity against NGP5B of mouse sera obtained at prime (P), boost 1–3 (B1-B3), three weeks post-B3 (day 0) and at the endpoint (43 dpi) from α1,3GalT-KO mice vaccinated with NGP5B, NGP5B+CpG, CpG, or PBS. (B-C) Antibody isotyping (IgG1, IgG2a, IgG2b, IgG3, and IgE) of mouse sera obtained following boost 3 (B3) and at the experimental endpoint (43 dpi). Statistical analysis for A-C: Two-way ANOVA with Dunnett’s multiple comparison test: (*), *P*<0.05; (****), *P*<0.0001. (D) Percentage of lysis of *L*. *major-luc* metacyclic promastigotes incubated with sera from mice immunized with NGP5B, NGP5B+CpG, CpG or PBS. Control dead parasites: 10^6^ *L*. *major*-*luc* metacyclic promastigotes, heat-killed at 100^°^C for 10 min, followed by 30-min incubation at RT with PI. Control live parasites: 10^6^ *L*. *major*-*luc* metacyclic promastigotes in DMEM (no FBS) without any treatment. C, mouse serum with active complement; iC, mouse serum with heat-inactivated complement; NMS, normal (non-infected) mouse serum. One-way ANOVA with Tukey’s multiple comparisons (compared with NMS control): ns, non-significant; (**), *P*<0.01; (***), *P*<0.001. (A-D) Error bars indicate S.E.M. of triplicate determinations.

To determine and characterize the humoral immune response produced by NGP5B and NGP5B+CpG, sera from all groups were collected 3 days after each immunization (P and B1-3), three weeks after last immunization, and at the endpoint (Fig 4A). First, the production of specific anti-NGP5B IgG antibodies was analyzed by chemiluminescent ELISA using NGP5B as antigen. As expected, high levels of specific IgG antibodies against NGP5B were observed in mice immunized with NGP5B or NGP5B+CpG, when compared to PBS or CpG alone. The high levels of anti-NGP5B antibodies were maintained until the experimental endpoint (Fig 5A). Furthermore, murine immunoglobulin isotypes (IgG1, IgG2a, IgG2b, IgG3, and IgE) specific against NGP5B were analyzed by ELISA (Fig 5B and 5C). At the end of the immunization (B3), mice immunized with NGP5B+CpG showed the following rank of IgG subclasses: IgG1>IgG2b>IgG2a>IgG3. Mice immunized with NGP5B alone, on the other hand, showed a slightly different rank of IgG subclasses: IgG1>IgG3>IgG2a>IgG2b>IgE (Fig 5B). Moreover, at the experimental endpoint (43 dpi), the levels of IgG1, IgG2a, IgG2b and IgG3 increased in mice immunized with NGP5B+CpG. In contrast, in the NGP5B-immunized group we observed an increase in IgG1, and decrease in IgG3 and IgG2a following *L*. *major-luc* challenge (Fig 5C). These results indicate that vaccination with NGP5B or NGP5B+CpG can stimulate production of specific IgG isotypes. Furthermore, no increase in the levels of IgE was observed following vaccination with NGP5B or NGP5B+CpG, or at the experimental endpoint (43 dpi).

### Anti-NGP5B antibodies have lytic activity against *L*. *major* metacyclic promastigotes

The lytic activity of anti-NGP5B antibodies elicited by animals vaccinated with NGP5B or NGP5B+CpG was investigated by incubating immunized mice sera with *L*. *major-luc* metacyclic promastigotes. As observed in Fig 5D, NGP5B+CpG (*P<*0.001) and NGP5B (*P*<0.01) antisera caused lysis in 60% and 44% of *L*. *major-luc* metacyclic promastigotes, respectively, when the complement was heat inactivated. Conversely, when complement in these antisera was active, we observed no significant parasite lysis, indicating that somehow active complement interferes with the parasite lysis. Therefore, mice immunized with NGP5B or NGP5B+CpG produced antibodies that are lytic in a complement-independent manner, suggesting a mechanism similar to lytic anti-α-Gal antibodies elicited against *Trypanosoma cruzi* trypomastigotes GPI-mucins [25, 44].

### Anti-NGP5B response is specific against terminal α-Gal residues

**Fig 6:**
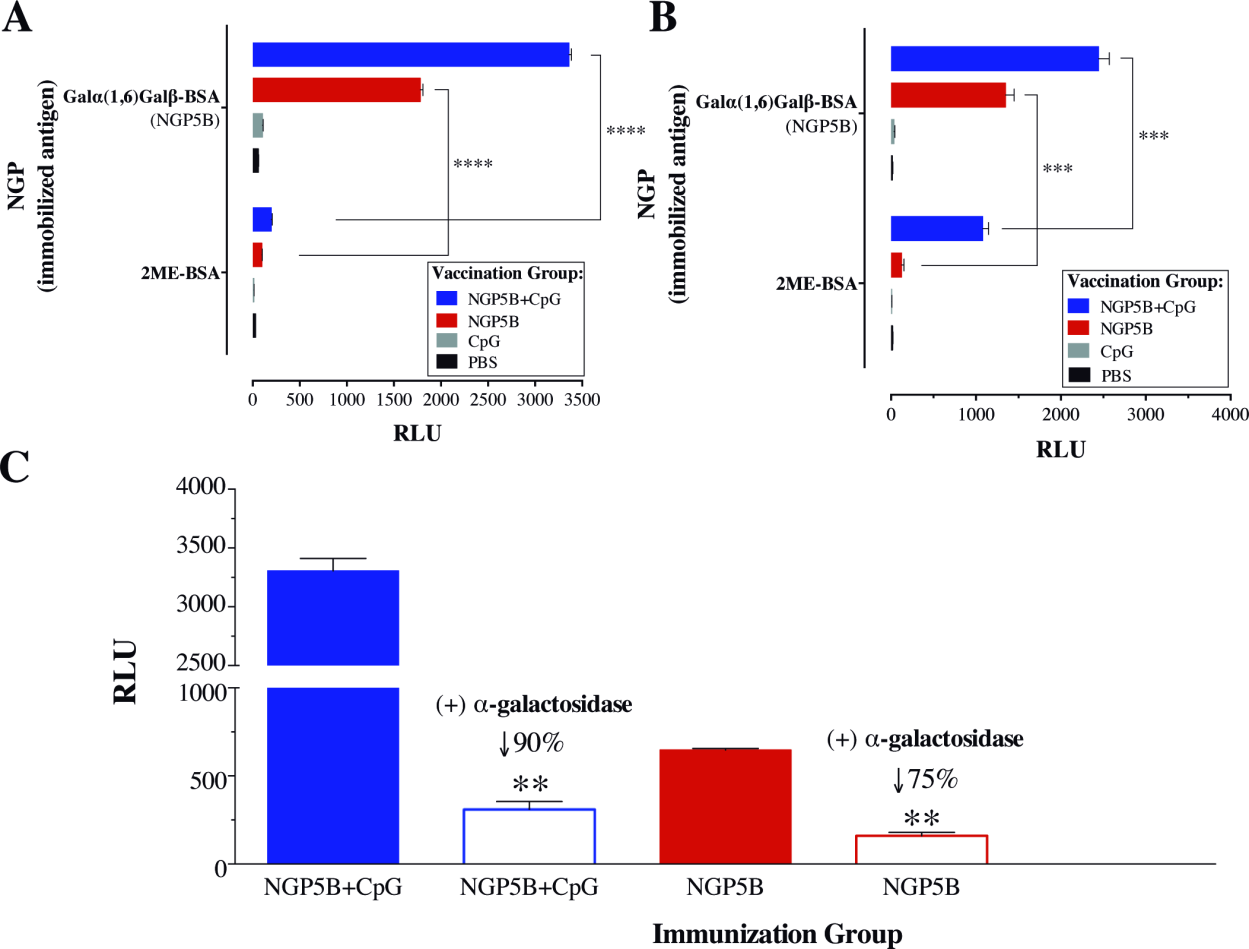
Anti-NGP5B antibody response is specific against terminal α-Gal residues. (A-B) Chemiluminescent ELISA reactivity of mouse serum pools obtained at Boost 3 (n = 6) and endpoint (n = 3) from α1,3GalT-KO mice vaccinated with NGP5B, NGP5B+CpG, CpG, or PBS. Immunized groups are indicated in the legend and antigens on the microplate are shown in the Y-axis. (C) Chemiluminescent ELISA reactivity of mouse serum obtained at Boost 2. NGP5B (125 ng/well) was treated or not with green-coffee bean α-galactosidase. One-way ANOVA (compared with untreated sample): (**), *P<*0.01; (***), *P*<0.001; (****), *P*<0.0001. (A-C) Error bars indicate S.E.M. of triplicate determinations.

The maleimide derivative succinimidyl 4-(*N*-maleimidomethyl)cyclohexane-1-carboxylate (SMCC) cross-linker commonly used for the conjugation of carbohydrates and peptides to BSA was reported to elicit high levels of anti-linker antibodies [45]. To detect antibodies against the maleimide cross-linker used in our NGPs, maleimide-derivatized BSA (Fig 1A) was blocked with 2-ME to give rise to 2ME-BSA [46]. The specificity of anti-NGP5B and anti-linker antibodies of sera (3 weeks post-B3 and endpoint) from mice immunized with NGP5B, NGP5B+CpG, CpG, or PBS was analyzed by chemiluminescent ELISA. As indicated in Fig 6A, sera from NGP5B- and NGP5B+CpG-immunized mice (3 weeks post-B3) had a very strong and specific antibody reactivity (*P*<0.0001) against NGP5B antigen and a very weak reactivity against the linker in 2ME-BSA antigen. However, we observed that at the endpoint of the experiment (Fig 6B), contrary to NGP5B, NGP5B+CpG elicited a considerable antibody response against 2ME-BSA. Next, NGP5B was treated or not with α-galactosidase, overnight at 37^°^C, to remove terminal α-Gal residues to confirm the main specificity of anti-NGP5B antibodies. In comparison with untreated groups, we observed a significant (*P*<0.01) decrease in the reactivity against NGP5B+CpG (90%) and NGP5B (75%) (Fig 6C). This result indicates that most antibodies produced against NGP5B an NGP5B+CpG recognize the terminal α-Gal residue in the NGP.

### NGP5B induces a protective Th1 cellular immune response

**Fig 7:**
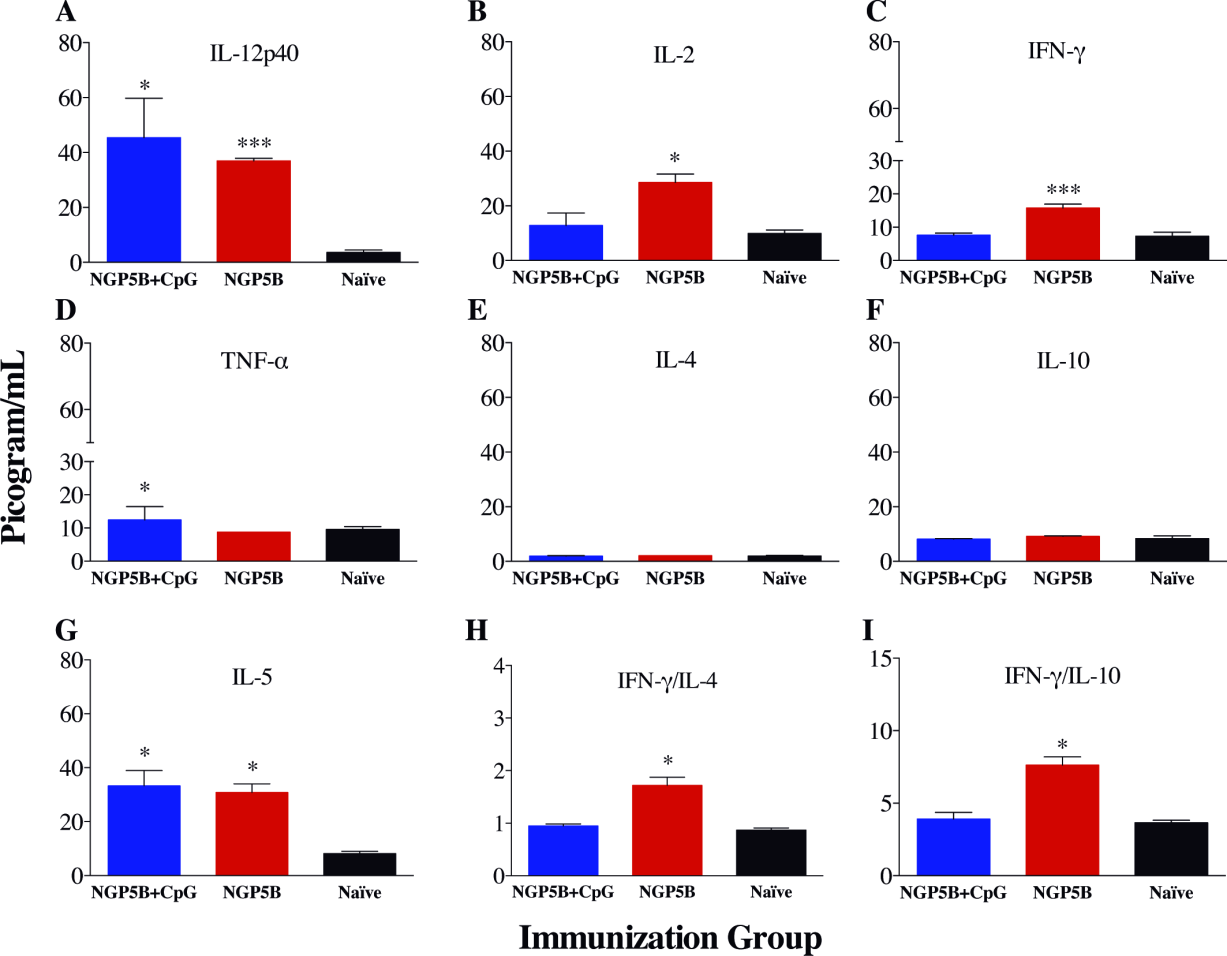
Serum cytokine profile of NGP5B and NGP5B+CpG immunizations. (A-D) Th1 cytokines IL-12p40, IL-2, IFN-γ, and TNF-α. (E-G) Th2 cytokines IL-4, IL-10, and IL-5. (H) IFN-γ/IL4 ratio. (I) IFN-γ/IL10 ratio. Two-tailed unpaired Student’s *t*-test (compared with Naïve group): (*), *P*<0.05; (***), *P*<0.0001. (A-I) Error bars indicate S.E.M. of triplicate determinations.

To study the cellular immune response induced by NGP5B and NGP5B+CpG vaccination in α1,3GalT-KO mice, a panel of Th1, Th2, and Th17 cytokines (IL-12p40, IL-2, IFN-γ, TNF-α, IL-5, IL-4, IL-10, IL-6, and IL-17) were analyzed in the sera of immunized mice before challenge with *L*. *major-luc* three weeks after last immunization (day 0; Fig 4A). Significant higher levels of Th1 cytokines: IL-12p40 (*P*<0.001), IL-2 (*P*<0.05), and IFN-γ (*P*<0.001) (Fig 7A–7D); and Th2 cytokine IL-5 (*P*<0.05) (Fig 7G) were observed in NGP5B-immunized group in comparison with control (naïve) group. Furthermore, we detected a significant increase in the IFN-γ/IL-4 (*P*<0.05) and IFN-γ/IL-10 (*P*<0.05) ratios in mice vaccinated with NGP5B (Fig 7H and 7I). A lower induction of proinflammatory cytokines was observed upon NGP5B+CpG immunization. Only IL-12p40 (*P*<0.05) and TNF-α (*P*<0.05) were significantly increased in that vaccinated group. On the other hand, we did not detect significant differences for IL-10, IL-4, IL-17, and IL-6 regulatory cytokines in comparison with control group (Fig 7E and 7F, and S1 Fig) in both NGP5B and NGP5B+CpG. In summary, these results suggest that animals immunized with NGP5B showed strong protective Th1 cellular immune response against *L*. *major* infection.

### Vaccination with NGP5B induces a robust T-cell response

**Fig 8:**
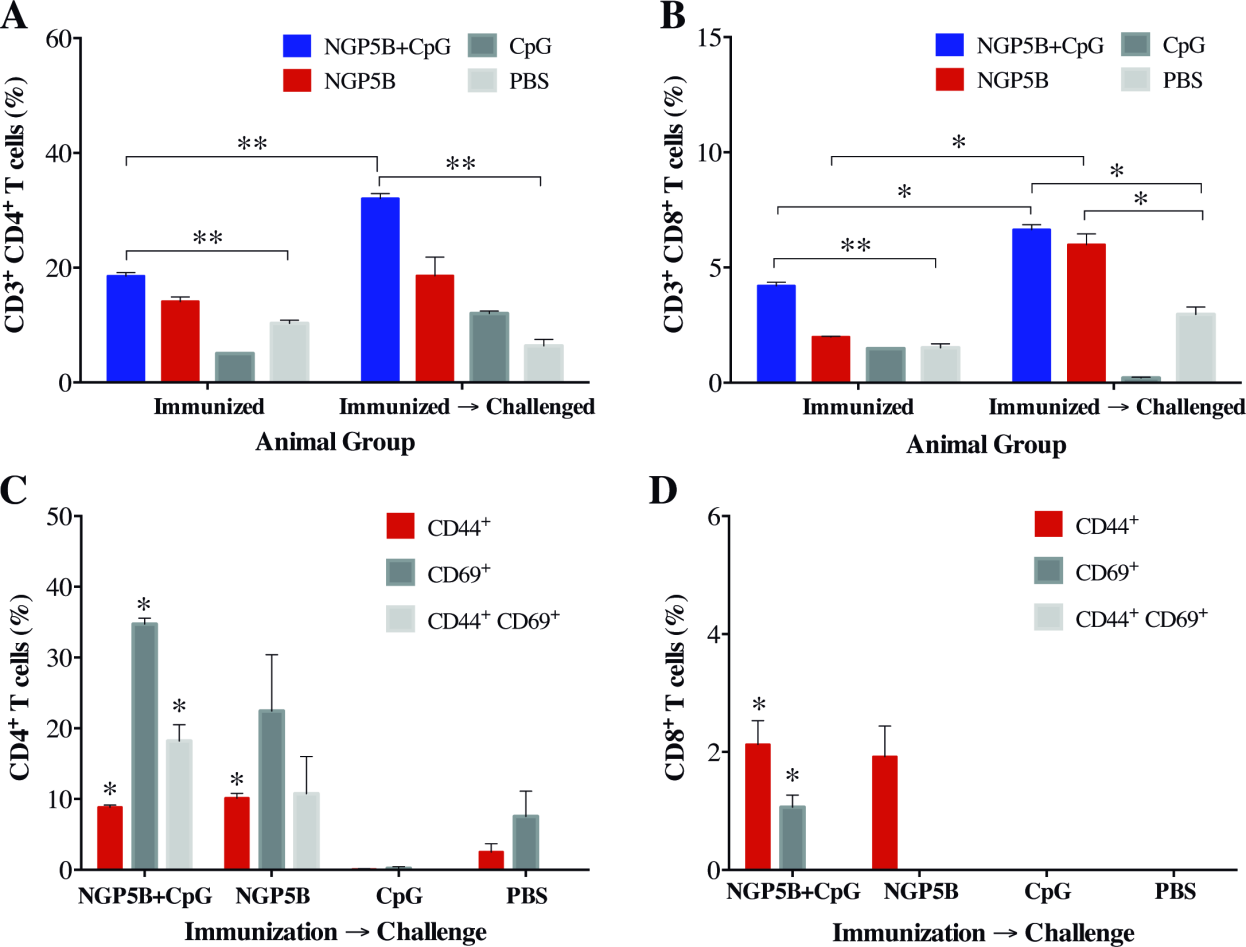
Vaccination with NGP5B or NGP5B+CpG induces antigen-specific CD4^+^ and CD8^+^ memory T cell after *L*. *major* challenge. (A and B) Percentage of antigen specific CD3^+^CD4^+^ and CD3^+^CD8^+^ T cells, respectively, from splenocytes of mice 3 weeks post-last immunization and at the endpoint (immunized→challenged). Splenocytes were cultured and stimulated *in vitro* with 20 μg/ml of antigen. (C) Splenocytes stimulated and gated on CD4^+^CD44^+^, CD4^+^CD69^+^, and CD4^+^CD44^+^CD69^+^ T cell populations from immunized-challenged group, for the percentage of activated CD4^+^ T cells in mice vaccinated with NGP5B or NGP5B+CpG. (D) Splenocytes stimulated and gated on CD8^+^CD44^+^, CD8^+^CD69^+^, and CD8^+^CD44^+^CD69^+^ T cell populations from immunized-challenged group, for the percentage of activated CD8^+^ T cells in mice vaccinated with NGP5B or NGP5B+CpG. Statistical analysis for A-D: Two-tailed unpaired multiple Student’s *t*-test: (*), *P*<0.05; (**), *P*<0.01. (A-D) Error bars indicate S.E.M. of triplicate determinations.

We first assessed the antigen-specific response of CD4^+^ and CD8^+^ T cells, at three weeks after last immunization (pre-challenge) and at the endpoint of the experiment (post-challenge). We also evaluated the levels of activated CD69^+^ and memory CD44^+^ T cells in immunized-challenged animals. NGP5B- and NGP5B+CpG-immunized mice developed a high expression of antigen-specific CD4^+^ and CD8^+^ T cell response, with a significant increase in the post-challenge time-points as compared to the control mice, CpG or PBS stimulated with NGP5B or NGP5B+CpG antigen (Fig 8A and 8B). Interestingly, after mice were challenged with *L*. *major*, a significant upregulation of memory CD44^+^CD4^+^ T cells and CD44^+^CD8^+^ T cells was found in both NGP5B (*P*≤0.05) and NGP5B+CpG (*P*≤0.05) groups (Fig 8C and 8D), together with high expression levels of CD4^+^CD69^+^ T cells (Fig 8C). These findings demonstrate that vaccination with NGP5B and NGP5B+CpG in α1,3GalT-KO mice can induce a robust CD4^+^ T cell response, accompanied by a CD8^+^ T cell response, both necessary for the protection against *L*. *major*.

## Discussion

Currently, leishmaniasis is endemic in 90 countries, with more than 2 million new cases per year, and a worldwide incidence of 350 million people at risk of infection [2, 3]. CL is the most common form of the disease with an estimated 0.7 to 1.2 million annual cases, representing 50–75% CL new cases [2]. Despite current advances [47–51], there is no human vaccine available that confers sterile protection against this neglected disease [52]. Nevertheless, previous studies have shown that a vaccine against CL is a viable and feasible approach [53]. Thus, a preventive CL vaccine will have a great public health impact in terms of treatment and preventing disease dissemination in conflict regions [3]. These facts underscore the urgent need for the development of a fully protective vaccine against CL. Here, by targeting α-Gal glycotopes abundantly expressed on the *L*. *major* surface [10, 11], we evaluated the first synthetic α-Gal-containing NGP (Galα(1,6)Galβ-BSA or NGP5B) against *L*. *major* infection in the α1,3GalT-KO mouse model.

First, we evaluated a series of eight synthetic α-Gal-containing NGPs as potential biomarkers in serum samples from patients infected with *L*. *major* from Saudi Arabia. Three highly differential biomarkers were selected (NGP17B, NGP12B, and NGP5B) and investigated as potential vaccine candidates against *L*. *major* infection in a murine model. We observed high levels of anti-α-Gal antibodies produced in α1,3GalT-KO mice vaccinated with NGP12B and NGP17B, offering partial protection against *L*. *major* infection. More importantly, NGP5B offered a significant, partial protection against the infection with a strong humoral response. To increase the protection against *L*. *major* infection, the CpG-ODN adjuvant in combination with NGP5B (NGP5B+CpG) was also evaluated. A robust antibody response was observed in both vaccinated groups (NGP5B and NGP5B+CpG), when compared to PBS and CpG control groups. Previously, Yilmaz *et al*. (2014) studied a synthetic α-Gal epitope (Galα(1,3)Galβ(1,4)GlcNAc) as vaccine candidate for *Plasmodium* infection [27]. Interestingly, they detected high levels of IgG1, IgG2b, and IgG3, and little or no circulating IgG2a in immunized α1,3GalT-KO mice [27]. Similarly, our NGP5B-immunized (noninfected) mice also showed high levels of IgG1 and IgG3, and lower levels of IgG2a and IgG2b subclasses. As for the NGP5B+CpG-immunized noninfected group, high levels of IgG1, IgG2b, and IgG2a and low levels of IgG3 and IgE were observed. The CpG adjuvant is known to increase murine B cells to produce IgG2a, IgG2b, and IgG3 isotypes [54]. Taken together, these findings indicate that NGP5B+CpG, and to a slightly lesser extent NGP5B, induces a strong IgG response associated with protection against *L*. *major* infection.

Consequently, the study of lytic parasite-specific anti*-*α*-*Gal antibodies is also of great interest in *L*. *major* infection as we anticipate that, similar to Chagas disease [22, 25, 29], these antibodies could have a protect role against infection. Here, we observed that sera from α1,3GalT-KO mice immunized NGP5B or NGP5B+CPG had a significant complement-independent lytic activity towards infective metacyclic promastigote forms of the parasite. Brittingham *et al*. (1985) [55] have previously demonstrated that *Leishmania* is able to exploit the opsonic effects of complement while avoiding complement-mediated lysis. The expression of gp63 accelerates the change of C3b to its inactive form, reducing the fixation of terminal complement components such as C5 and C7, and the formation of the membrane attack complex, resulting in a resistance to complement-mediated lysis [55]. Furthermore, previous work in the metacyclic promastigote form of the parasite demonstrate an increased resistance to complement-mediated lysis in comparison to the promastigote form of the parasite [56]. Our study provides evidence that immunization with NGP5B or NGP5B+CPG can elicit anti-α-Gal antibodies with lytic, complement-independent activity against *L*. *major-luc* metacyclic promastigotes, which may serve as one of the mechanisms conferring protection against infection.

To successfully control CL infection, a Th1-mediated immunity is desired [57, 58]. We observed that NGP5B induced a predominantly Th1-mediated cellular immune response in α1,3GalT-KO mice, with an increase in IL-12p40, IL-2, and IFN-γ, which are related to the protection against CL. On the other hand, NGP5B+CpG induced mainly IL-12p40 and TNF-α. Although not statistically significant, there was a trend of higher IL-12p40 level in the NGP5B+CpG-immunized group in comparison to the group immunized with NGP5B alone. It has been recently reported that CpG-ODN can upregulate the secretion of IL-12 by the help of inflammatory monocytes [59]. It is known that macrophages are primarily involved in the destruction of intracellular parasites such as *L*. *major* by the secretion of IL-12, causing the differentiation of CD4^+^ T cells into Th1 cells and the production IFN-γ and IL-2 cytokines [60–62]. The production of IL-12 and IFN-γ activates the up-regulation of nitric oxide in macrophages producing an oxidative burst that subsequently kills the parasite. Additionally, no significant increase in IL-10 and IL-4 cytokines were observed, which are associated with disease progression through suppression of macrophage activation [60–62]. On the contrary, significant higher IFN-γ/IL-4 and IFN-γ/IL-10 ratios, which are indicative of a favorable outcome in CL infection [57], were observed in the animals vaccinated with NGP5B. Interestingly, recruitment and maturation of eosinophils to the site of *L*. *major* infection is modulated by the production of IL-5 and studies have shown that eosinophils play a role in parasite clearance by suppressing the developing lesion and having antimicrobial activity against *L*. *mexicana* and *L*. *donovani* [63]. Therefore, induction of this cytokine, observed here by immunization with NGP5B, could be beneficial for the control of *L*. *major* infection in α1,3GalT-KO mice.

The activation and expansion of T cells are fundamental to mount an adaptive immune response for long-term protection against *L*. *major* infection and essential in vaccine efficacy optimization [64, 65]. Therefore, in our study, the cellular immune response was determined by the percentage of activated CD4^+^ and CD8^+^ T cells in both immunized non-challenged, and immunized challenged mice. Additionally, CD44 and CD69 expression was measured for T cell activation before and after infection with *L*. *major*. First, we observed a robust antigen specific CD4^+^ T cell response as well as a CD8^+^ T cell response before and after infection. Interestingly, after infection (43 dpi), a high proportion of antigen specific CD4^+^ T cells expressing CD44 and CD69 were observed in both NGP5B and NGP5B+CPG mice. Moreover, a small population of CD8^+^ T cells expressing CD44 was also observed. This may suggest that antigen-dependent T-cell stimulation was still in course [66]. Altogether, these results suggest that NGP5B or NGP5B+CPG vaccination can elicit strong T cell immunity, since both CD4^+^ and CD8^+^ T cells are important in controlling parasite burden against *L*. *major* infection [57].

Previously, the immunogenicity of three different maleimide derivatives linkers was investigated [45]. Interestingly, a high level of anti-linker antibodies was found against maleimide-derivative linkers containing an additional aliphatic or aromatic ring [45]. In this study, we found that NGP5B-vaccinated mice had a specific and strong antibody response against the terminal α-Gal epitope in NGP5B, and not against the linker when using 2-ME-BSA as antigen [46]. However, we observed that the NGP5B+CPG group had a significant antibody response against 2-ME-BSA at the experimental endpoint (43 dpi), suggesting that the *L*. *major* infection might be eliciting antibodies that can cross-react with the linker. Nevertheless, for future glycan conjugation to the carrier protein, a smaller and more flexible linker could be used to achieve a more specific immune response against the glycotope [46].

Carbohydrates have been proposed as potential targets for vaccines against *Leishmania* spp. [32, 67]. Vaccination with *Leishmania* lipophosphoglycan (LPG), one of the major components of the glycocalyx of the parasite, revealed contradictory results [68–70]. For instance, LPG purified from *L*. *donovani* failed to protect against *L*. *major* infection in BALB/c mice [68]. Martinez Salazar et al. [71] suggested that *L*. *mexicana* LPG vaccination induced upregulation of programmed cell death protein 1 (PD-1), leading to functional CD8^+^ T cell inactivation and, consequently, disease progression. Conversely, Pinheiro et al. [70] demonstrated that intranasal vaccination with *L*. *amazonensis* LPG provided protection in BALB/c mice against subsequent parasite challenge. Additionally, a synthetic glycovaccine containing fragments of the phosphoglycan moiety of LPG or proteophosphoglycan (PPG) from *L*. *mexicana* exerted a protective effect in a natural sand fly infection model of CL caused *L*. *mexicana* [72]. In the present study, we explored NGPs containing non-reducing, terminal α-Gal epitopes similar to those found in Type-II GIPL-2 and GIPL-3 of *L*. *major* [10, 14], as preventive CL vaccines. For instance, the NGP5B glycotope, Galα(1,6)Galβ, is analogous to the GIPL-3 glycotope, Galα(1,6)Galα-R, and showed to be highly reactive to sera from active CL infections and partially protective in the α1,3GalT-KO mouse model. Evaluation of purified GIPL-3 or its synthetic terminal α-Gal-containing glycotope(s) covalently coupled to a carrier protein as vaccine candidates may provide evidence whether protective anti-α-Gal antibodies against NGP5B cross-react with the native glycotope. Furthermore, we hypothesize that the α-Gal-terminating NGP5B, NGP12B, and NGP17B, evaluated here as vaccine candidates against *L*. *major*, and eventually additional α-Gal-terminating NGPs may protect against other *Leishmania* species that express Type-II GIPLs (e.g., *L*. mexicana) [73] or Type-II-like GIPLs (e.g., *L*. *brasiliensis* and *L*. *infantum*) [74]. We also speculate that similar or distinct α-Gal-terminating glycotopes could be expressed in other *Leishmania* glycoconjugates (e.g., LPG and PPG) and could play a protective role against leishmaniasis. In this regard, the evaluation of current and additional α-Gal-terminating NGPs as vaccine candidates against other *Leishmania* species will be pursued in future studies.

Previous reports have shown that the sand fly saliva plays a critical role in the establishment and maintenance of *Leishmania* spp. in the mammalian host, as well as in the pathogenesis of leishmaniasis and immune responses against the parasite (reviewed in [75, 76]). Accordingly, the efficacy of vaccines against *Leishmania* spp. might be considerably influenced by the parasite challenge procedure, either by needle or sand fly bite. For instance, Peters et al. [77] showed that the protective effect of a complex *L*. *major* vaccine preparation in mice that were initially needle-challenged could be subsequently abrogated by a sand fly-mediated infection. On the other hand, Oliveira et al. [78] demonstrated that the protective effect of *Phlebotomus duboscqi* uninfected sand fly bites in rhesus macaques. First, they observed that only 30% of animals exposed to uninfected sand fly bites followed by vector-transmitted *L*. *major* challenge, exhibited ulcerated lesions, in comparison to 70% of naïve animals. Furthermore, macaques immunized with a *P*. *duboscqi*-derived recombinant salivary protein (PdSP15), followed by sand fly-challenge with *L*. *major*, showed a significant reduction in disease burden, resulting from an early Th1-mediated anti-parasitic protective immune response involving CD4^+^IFN-γ^+^ T cells. Moreover, besides salivary proteins, *Leishmania* also releases large quantities of promastigote secretory gel (PSG) within the sand fly, a glycoconjugate that is regurgitated along with parasites and is important for establishment of the infection in skin [79]. In summary, these and other studies (reviewed in [75, 76]) highlight the paramount importance of the natural sand fly-transmitted model of infection in the assessment of efficacy of any potential vaccine against *Leishmania* spp. To this end, the efficacy of current and novel α-Gal-terminating NGP vaccine candidates will eventually be evaluated in α1,3GalT-KO mice by sand fly-mediated challenge.

In conclusion, here we have demonstrated that the α-Gal-terminating NGP5B vaccine candidate, in the presence or not of CpG adjuvant, conferred partial but significant protection against *L*. *major* infection in mice, by eliciting a robust B- and T-cell mediated immune response through lytic, complement-independent anti*-*α*-*Gal antibodies and protective Th1 cytokines.

## Supporting information

S1 Fig Levels of IL-6 and IL-17 in the mouse sera following immunization with NGP5B or NGP5B+CpG. IL-6 and IL-17 were analyzed in the sera of immunized mice three weeks after last immunization (B3), prior to challenge with *L*. *major-luc* (day 0, Fig 4A), as described in Materials and Methods. No significant difference was observed between NGP5B or NGP5B+CpG group and Naïve group using two-tailed unpaired Student’s *t*-test. Error bars indicate S.E.M. of triplicate determinations.
(TIF)
