# Supplementary figures and images for: An α-Gal-containing neoglycoprotein-based vaccine partially protects against murine cutaneous leishmaniasis caused by Leishmania major
Source: PLoS Negl Trop Dis. 2017 Oct 25;11(10):e0006039. doi: 10.1371/journal.pntd.0006039 (PMC5673233; doi:10.1371/journal.pntd.0006039)

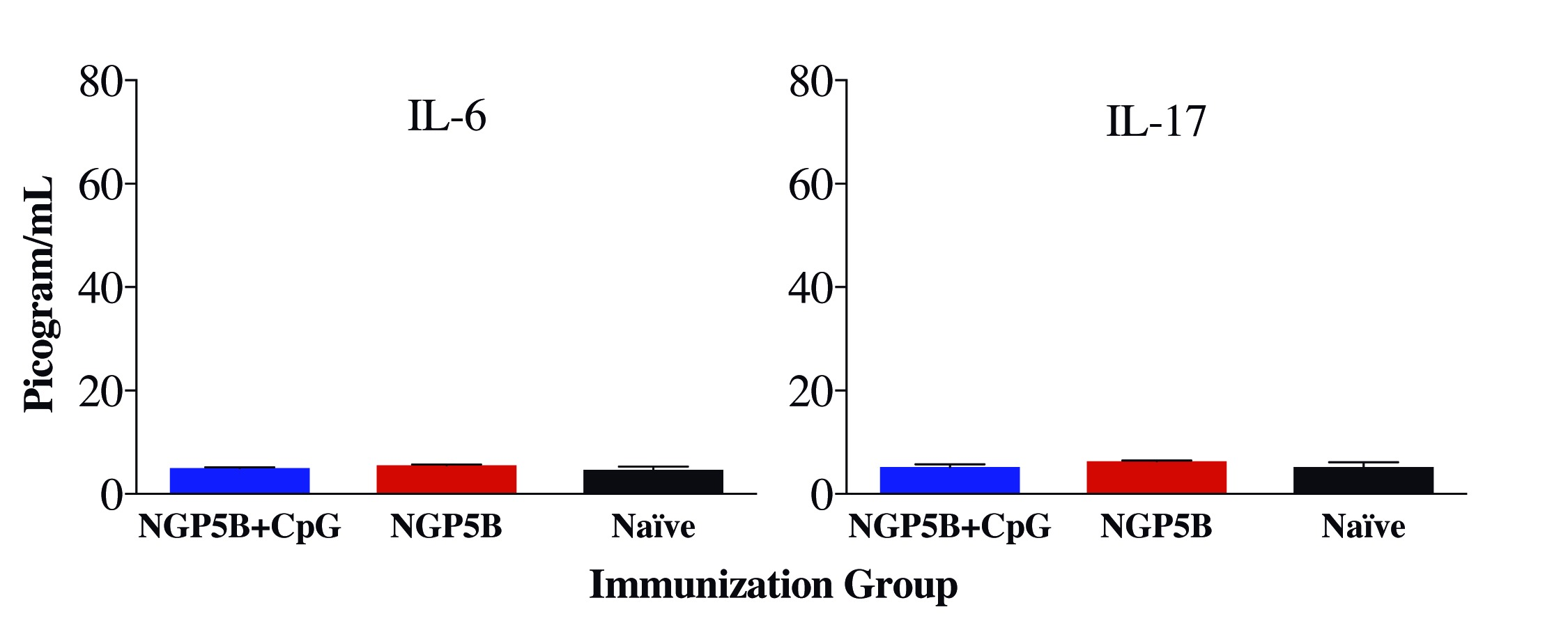

Supplement: S1 Fig — IL-6 and IL-17 were analyzed in the sera of immunized mice three weeks after last immunization (B3), prior to challenge with L. major-luc (day 0, Fig 4A), as described in Materials and Methods. No significant difference was observed between NGP5B or NGP5B+CpG group and Naïve group using two-tailed unpaired Student’s t-test. Error bars indicate S.E.M. of triplicate determinations. (TIF) [file pntd.0006039.s001.tif]
